# Characteristics and Absolute Survival of Metastatic Colorectal Cancer Patients Treated With Biologics: A Real-World Data Analysis From Three European Countries

**DOI:** 10.3389/fonc.2021.630456

**Published:** 2021-03-05

**Authors:** Katja A. Oppelt, Josephina G. Kuiper, Ylenia Ingrasciotta, Valentina Ientile, Ron M. C. Herings, Michele Tari, Gianluca Trifirò, Ulrike Haug

**Affiliations:** ^1^Department of Clinical Epidemiology, Leibniz Institute for Prevention Research and Epidemiology – BIPS, Bremen, Germany; ^2^PHARMO Institute for Drug Outcomes Research, Utrecht, Netherlands; ^3^Department of Biomedical and Dental Sciences and Morphofunctional Imaging, University of Messina, Messina, Italy; ^4^Caserta Local Health Unit, Caserta, Italy; ^5^Faculty of Human and Health Sciences, University of Bremen, Bremen, Germany

**Keywords:** colorectal cancer, biologics, survival, Europe, real-world data

## Abstract

**Introduction:** Biologics were approved for the treatment of advanced colorectal cancer (CRC) based on favorable benefit-risk-assessments from randomized controlled trials (RCTs), but evidence on their use in the real-world setting is scarce. Based on descriptive analyses we therefore aimed to assess characteristics and survival of CRC patients treated with biologics using large healthcare databases from three European countries (Netherlands, Italy, Germany).

**Methods:** We included CRC patients treated with a biologic in 2010 or 2014 and characterized them regarding age, sex, comorbidities, and absolute survival.

**Results:** Among 4,758 patients, the mean age ranged from 64.8 to 66.8 years, the majority was male, and comorbidities used as exclusion criteria in RCTs were coded in up to 30% of these patients. The proportion of bevacizumab users decreased between 2010 (72–93%) and 2014 (63–85%). In 2014, the absolute 12-month survival in new users was 64% (95% CI 51–77%), 56% (30–80%), and 61% (58–63%) in the Dutch, Italian, and German database, respectively, varying by age and comorbidity.

**Conclusions:** Our study suggests that in the real-world setting, CRC patients treated with biologics are older and less selected regarding comorbidities compared to patients in RCTs, potentially explaining the relatively low 12-month survival we found. Treatment decisions in the real-world setting may require careful evaluation given that the risk-benefit ratio may vary depending on age and co-existing conditions.

## Introduction

Several randomized controlled trials (RCTs) have shown that biologic drugs, called “biologic” because they are produced by living organisms, may improve survival in patients with advanced colorectal cancer (CRC) (1–4). One example is the pivotal study on bevacizumab published in 2004. Bevacizumab was one of the first biologic drugs developed for treating metastatic CRC. The study showed a 12-month survival of 74.3% for patients combining bevacizumab and chemotherapy, as compared to 63.4% for patients receiving chemotherapy alone (5). With respect to adverse events the incidence of grade 3 or 4 adverse events in the bevacizumab group was 10 percentage points higher (statistically significant difference) and the incidence of hospitalizations due to adverse events was five percentage points higher (5). A positive assessment of the risk-benefit ratio and confirmatory results from further RCTs led to the approval by the European Medicines Agency of bevacizumab for the treatment of advanced CRC in 2005 (6).

However, the risk-benefit ratio observed in clinical trials conducted under controlled conditions and in selected study populations is not necessarily similar to the risk-benefit ratio in the real-world setting. In particular, a poorer health status overall or a higher prevalence of certain comorbidities could negatively affect this ratio (7). Monitoring the use of these drugs in the real-world setting is thus urgently needed.

So far, available studies using real-world data such as administrative claims are often based on data from the United States (8–10). To our knowledge, real-world evidence based on routinely collected data on utilization of biologics in CRC patients from Europe is limited to two studies from the Czech Republic using data from a specific drug registry, one study from the Netherlands using data from a regional cancer registry, and one from Italy based on five regional cancer registries (11–14). Large claims or medical record databases from Europe have thus not been used for this purpose so far.

To shed further light on this topic, we aimed to explore the potential of large European healthcare databases for real-world monitoring of biologics in the treatment of CRC. Based on descriptive analyses we assessed the general characteristics, treatment patterns, and overall survival of patients using one or more of the three biologics available for CRC treatment during the study period, namely the vascular endothelial growth factor (VEGF)-inhibitor bevacizumab, slowing the growth of new blood vessels, and the two epidermal growth factor receptor (EGFR)-inhibitors cetuximab and panitumumab, inhibiting cell growth and division.

## Methods

### Data Sources

We conducted a retrospective cohort study based on healthcare databases from three European countries [Netherlands: PHARMO Database Network (PHARMO); Italy: Caserta Local Health Unit (Caserta LHU); Germany: German Pharmacoepidemiological Research Database (GePaRD)]. A detailed description of these databases is provided in the (Supplementary Material 1). In brief, the PHARMO is a population-based network of electronic healthcare databases currently covering over 6 million persons out of 17 million inhabitants of the Netherlands (15). It combines anonymous data from different primary and secondary healthcare settings in the Netherlands. For this study, we used data from the Hospital Database, the In-patient Pharmacy Database, and the Out-patient Pharmacy Database, linked on a patient level through validated algorithms.

Caserta LHU contains claims data from several databases since 2009. It covers ~1.2 million residents of Caserta (Italy) from 2009 to 2014. It includes, amongst others, information on drug dispensing in the outpatient setting, hospitalizations, outpatient diagnostic tests, and specialists' visits (16–19).

GePaRD is based on claims data from four statutory health insurance providers in Germany and currently includes information on ~25 million persons who have been insured with one of the participating providers since 2004 or later. In addition to demographic data, GePaRD contains information on drug dispensings, outpatient, and inpatient services and diagnoses (20).

### Study Design and Study Population

In each database, we aimed to include CRC patients exposed to biologics in 2014 (cohort 2014) and for comparison also CRC patients exposed to biologics in 2010 (cohort 2010, available in GePaRD and PHARMO only). Exposure to biologics was defined as at least one in- or outpatient dispensing of any relevant biologic (bevacizumab, cetuximab, panitumumab; see Supplementary Material 2 for a list of ATC codes) in the respective year. We selected the cohorts in a two-step process. First, we identified all persons with such a dispensing in the respective year and defined the day of their first dispensing as cohort entry. Second, we limited the cohorts to patients with a CRC diagnosis (PHARMO and GePaRD: ICD-10: C18-20; Caserta LHU: ICD-9: 153^*^, 154^*^). In GePaRD, a previously developed algorithm was used to identify CRC cases (21). We considered CRC diagnoses during a preobservation period of 1 year before and on the day of cohort entry. Comorbidities and the presence of metastases were identified by ICD-10 and ICD-9 codes, respectively, and database-specific algorithms. We defined the cohort exit as the end of follow-up or death, whichever came first.

### Data Analyses

We characterized the patients regarding age, sex, presence of codes for metastases, and length of follow-up. Furthermore, we assessed the prevalence of comorbidities during the preobservation period, which were defined as exclusion criteria in the pivotal study by Hurwitz et al., namely cardio-vascular diseases, ascites, metastases of the central nervous system, bleeding diatheses, and coagulopathy (see Supplementary Material 3 for a list of ICD-10 and ICD-9 codes) (5).

For each cohort, we determined the type of biologic drug leading to cohort entry as well as the number of different biologic drugs dispensed during a follow-up of 12 and 30 months. We used Kaplan-Meier survival analyses to describe absolute survival after cohort entry. We restricted these analyses to new users of biologics defined as persons without a dispensation of biologics in the 12 months before cohort entry. This helped to avoid the comparison of patients in different phases of treatment with biologics and also ensured a better comparability with clinical trials, which typically report the survival for persons initiating treatment with biologics. In GePaRD, we also assessed overall survival among new users stratified by age group and in the subgroup of new users with the above-mentioned comorbidities. In a subsample of GePaRD containing new users from one participating statutory health insurance which provides information on the number of cytostatic agents used in in- and outpatient chemotherapy, we assessed the number of new users having received chemotherapy within 30 days before or after cohort entry including the number of cytostatic agents used. All analyses were conducted in SAS (22).

## Results

### Characteristics of the Study Population

Overall, we identified 2,274 CRC patients exposed to biologics in 2010 and 2,484 patients in 2014. Table 1 shows the number of included patients and their characteristics stratified by database and year. GePaRD contributed the largest proportion of patients both in 2010 and 2014 (95%). The mean age ranged from 64.8 to 66.8 years and the proportion of males ranged from 53.2 to 65.3%. In the majority of patients, there were codes for metastases with some variation between databases. In the data from GePaRD and from Caserta LHU, there were codes for cardiovascular disease in more than 20% of patients, while this proportion was lower in PHARMO. Other comorbidities used as exclusion criteria in the study by Hurwitz et al. such as ascites were coded mainly in GePaRD among 0.6–3.3% of patients.

**Table 1:** Characteristics of colorectal cancer patients using biologics in 2010 and 2014.

	**2010**		**2014**		
	**GePaRD (Germany)**	**PHARMO (Netherlands)**	**GePaRD (Germany)**	**PHARMO (Netherlands)**	**Caserta LHU (Italy)**
Number of patients	2,162	112	2,362	73	49
Sex male [percent]	54.7^a^	62.5	53.2^a^	63.0	65.3
Mean age (SD) [years]	65.7 (10.0)	65.8 (8.9)	66.8 (10.4)	64.8 (9.0)	66.4 (11.9)
Median age (Q1, Q3) [years]	67 (60, 73)	68 (61, 72)	68 (60, 74)	65 (61, 72)	68 (58, 75)
<60 years [percent]	24.8	23.2	23.5	24.6	
60–75 years [percent]	60.0	58.9	56.0	61.6	
>75 years [percent]	15.4	17.9	20.5	13.7	
Presence of distant metastases [percent]	90.3	66.1	92.3	72.6	81.6
**Comorbidities [percent]**					
Cardio-vascular diseases	28.5	2.7	29.8	5.5	22.4
Ascites	1.9	0.0	3.3	0.0	2.0
CNS metastases	1.3	0.0	1.2	0.0	0.0
Bleeding diatheses	0.7	0.0	0.6	0.0	0.0
Coagulopathy	1.0	0.0	1.6	0.0	0.0

a *In one of the health insurances providing data of about 6 million insured persons to GePaRD, the proportion of women 50 years old or older is substantially higher as compared to the general population (32.1 vs. 22.5%). This explains the unexpected gender distribution among patients with CRC in GePaRD*.

### Type and Number of Biologics Used

Table 2 shows the type of biologic and the number of different biologics used during a follow-up period of 30 months. In GePaRD, the proportion of patients using bevacizumab and cetuximab decreased between 2010 and 2014: For bevacizumab, it decreased from 81% in 2010 to 76% in 2014, for cetuximab it increased from 39% in 2010 to 33% in 2014. During the same time, the proportion of patients using panitumumab increased from 20% in 2010 to 24% in 2014. In PHARMO, the proportion of patients using bevacizumab was 93% in 2010 and decreased to 85% in 2014. For cetuximab, the proportion was 2% (2010) and 4% (2014) and for panitumumab, it increased from 16% in 2010 to 29% in 2014. In the database from Caserta LHU (data from 2014 only), the proportion of patients using bevacizumab (82%) was similar to PHARMO in 2014, while for cetuximab, the proportion was 25% and thus similar to GePaRD in 2014.

**Table 2:** Type and number of biologics used by colorectal cancer patients.

	**2010**		**2014**		
	**GePaRD (Germany)**	**PHARMO (Netherlands)**	**GePaRD (Germany)**	**PHARMO (Netherlands)**	**Caserta LHU (Italy)**
	***N* = 2,162**	***N* = 112**	***N* = 2,362**	***N* = 73**	***N* = 49**
**Biologics used during 30 months of follow-up^a^ [percent]**					
Bevacizumab	80.5	92.9	76.3	84.9	81.6
Cetuximab	39.1	1.8	32.7	4.1	24.5
Panitumumab	19.7	16.1	24.1	28.8	6.1
**Number of different biologics used during 30 months follow-up [percent]**					
One	69.9	89.3	78.2	82.2	87.8
Two	23.3	10.7	19.4	17.8	12.2
More than two	6.8	0.0	2.4	0.0	0.0

a *Since use of multiple drugs per patient was possible, numbers may add up to more than 100%*.

Across all databases, 11–30% of patients received two or more different biologics relevant regarding CRC during 30 months of follow-up. In both years, this proportion was highest in GePaRD where it decreased from 30% in 2010 to 22% in 2014. Only patients from GePaRD received more than two different biologic drugs (7% in 2010 and 2% in 2014).

In the subsample of GePaRD containing new users from the (only) statutory health insurance that provides data on the number of cytostatic agents used in in- and outpatient chemotherapy (*n* = 2,417), 95% received chemotherapy within 30 days before or after cohort entry. Of these patients, 19% were treated with one cytostatic agent, 66% received two different cytostatic agents, and 15% received three or more different cytostatic agents.

### Description of Survival

**Figure 1:**
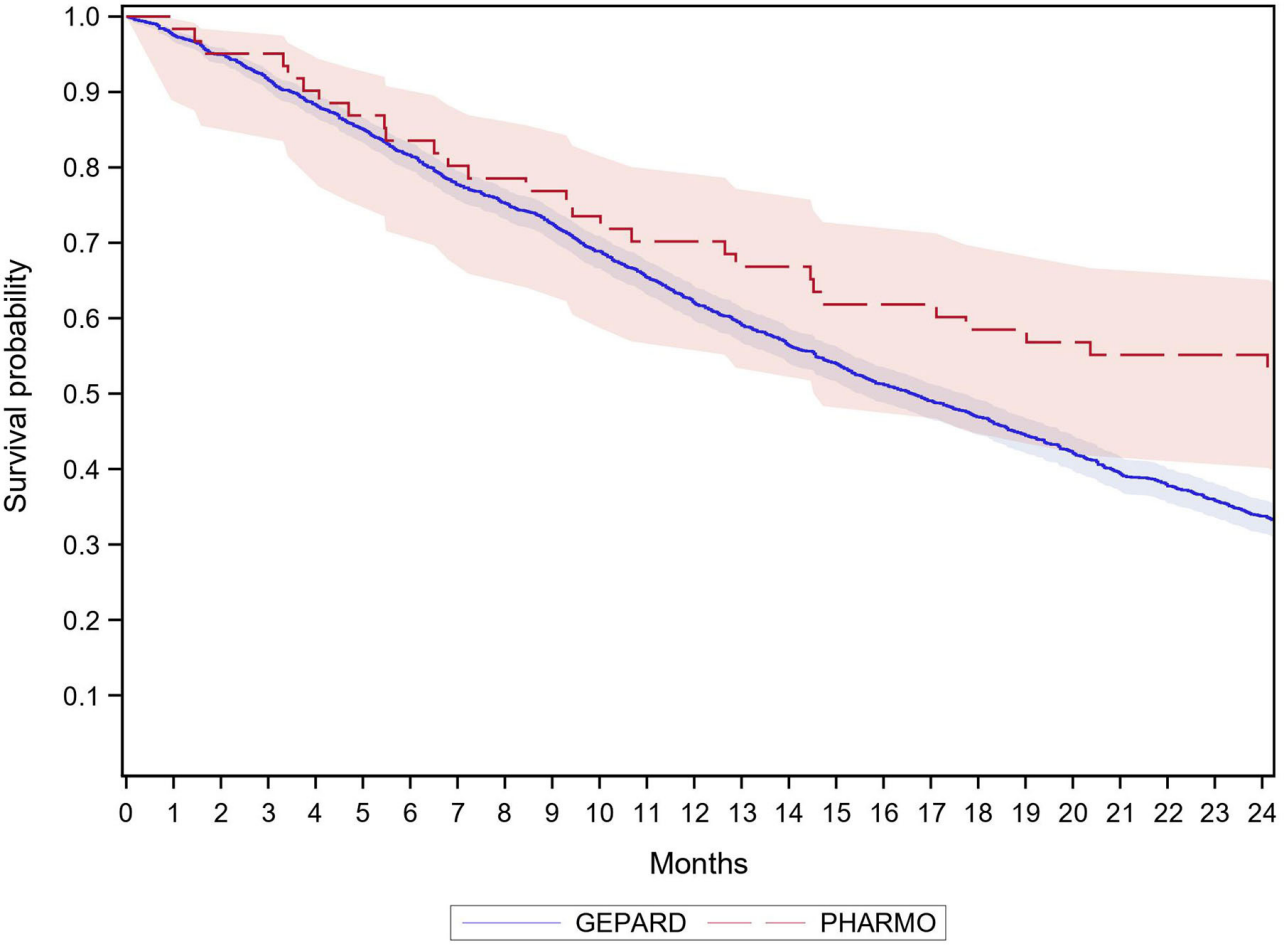
Survival of new users of biologics with colorectal cancer by database 2010.

**Figure 2:**
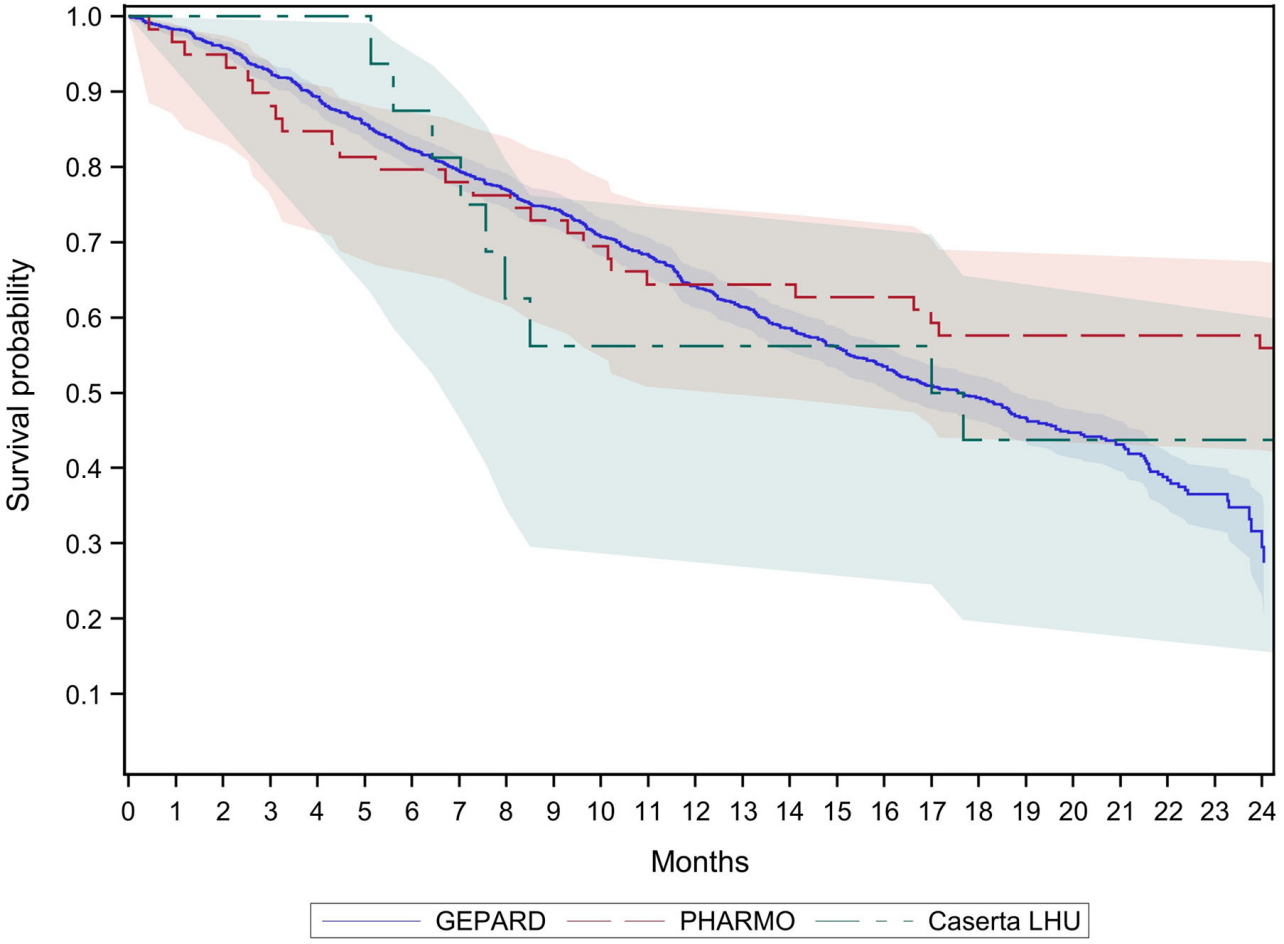
Survival of new users of biologics with colorectal cancer by database 2014.

Table 3 shows the absolute 12-month survival among CRC patients treated with biologics. In GePaRD, about 40% of the patients died within 12 months after cohort entry in both years. This applied to all patients as well as to new users of biologics, i.e., those without a dispensing of biologics in the 12 months before cohort entry. In the other databases, the point estimates of this proportion varied from 31 to 44% and had a large confidence interval that included the point estimates of GePaRD. As illustrated in Figures 1, 2, the probability of dying among new users of biologics increased to about 70% within 24 months in GePaRD and tended to be lower in PHARMO (with non-overlapping 95% confidence intervals, i.e., statistical significance at the 0.05 level). Compared to GePaRD, the probability of dying was also lower for patients from the Caserta LHU database, but the confidence intervals were large and included the point estimates observed in GePaRD and PHARMO.

**Table 3:** 12-month absolute survival of colorectal cancer patients using biologics overall (all databases) and stratified by age and presence of selected comorbidities (GePaRD only).

	**2010**		**2014**		
	**GePaRD (Germany)**	**PHARMO (Netherlands)**	**GePaRD (Germany)**	**PHARMO (Netherlands)**	**Caserta LHU (Italy)**
**Users of biologics, 12-month survival (95% CI) [percent]**					
All	60.4 (58.3–62.4)	65.2 (56.2–74.5)	60.7 (58.7–62.7)	63.0 (50.9–74.0)	63.3 (48.3–76.6)
**New users of biologics, 12-month survival (95% CI) [percent]**					
All	60.7 (58.4–63.0)	68.9 (55.7–80.1)	61.8 (59.1–64.5)	64.4 (50.9–76.5)	56.3 (29.9–80.3)
**Stratified by age in years**					
<60	62.5 (57.8–67.1)		67.8 (62.2–73.0)		
60–75	61.5 (58.5–64.5)		63.5 (59.9–67.1)		
>75	54.9 (48.8–61.0)		50.2 (44.0–56.4)		
**New users of bevacizumab, 12-month survival (95% CI) [percent]**					
All	59.5 (56.3–62.6)		62.1 (58.4–65.7)		
**Stratified by age in years**					
<60	60.2 (53.2–66.9)		71.2 (63.2–78.4)		
60–75	61.3 (57.2–65.3)		62.5 (57.5–67.4)		
>75	52.2 (44.2–60.0)		52.8 (44.8–60.7)		
**New users of biologics with selected comorbidities^a^, 12-month survival (95% CI) [percent]**					
All	56.4 (52.2–60.5)		56.9 (52.2–61.5)		
**Stratified by age in years**					
<60	50.0 (38.6–61.4)		53.2 (38.1–67.9)		
60–75	59.8 (54.5–65.0)		61.5 (55.5–67.2)		
>75	51.1 (42.4–59.7)		48.0 (39.0–57.1)		

a *We considered comorbidities that often led to exclusion of patients from randomized controlled trials investigating biologics in colorectal cancer patients: cardio-vascular diseases, ascites, CNS metastases, bleeding diatheses, and coagulopathy (for a list of the respective ICD-9 and ICD-10 codes see Supplementary Material 3)*.

**Figure 3:**
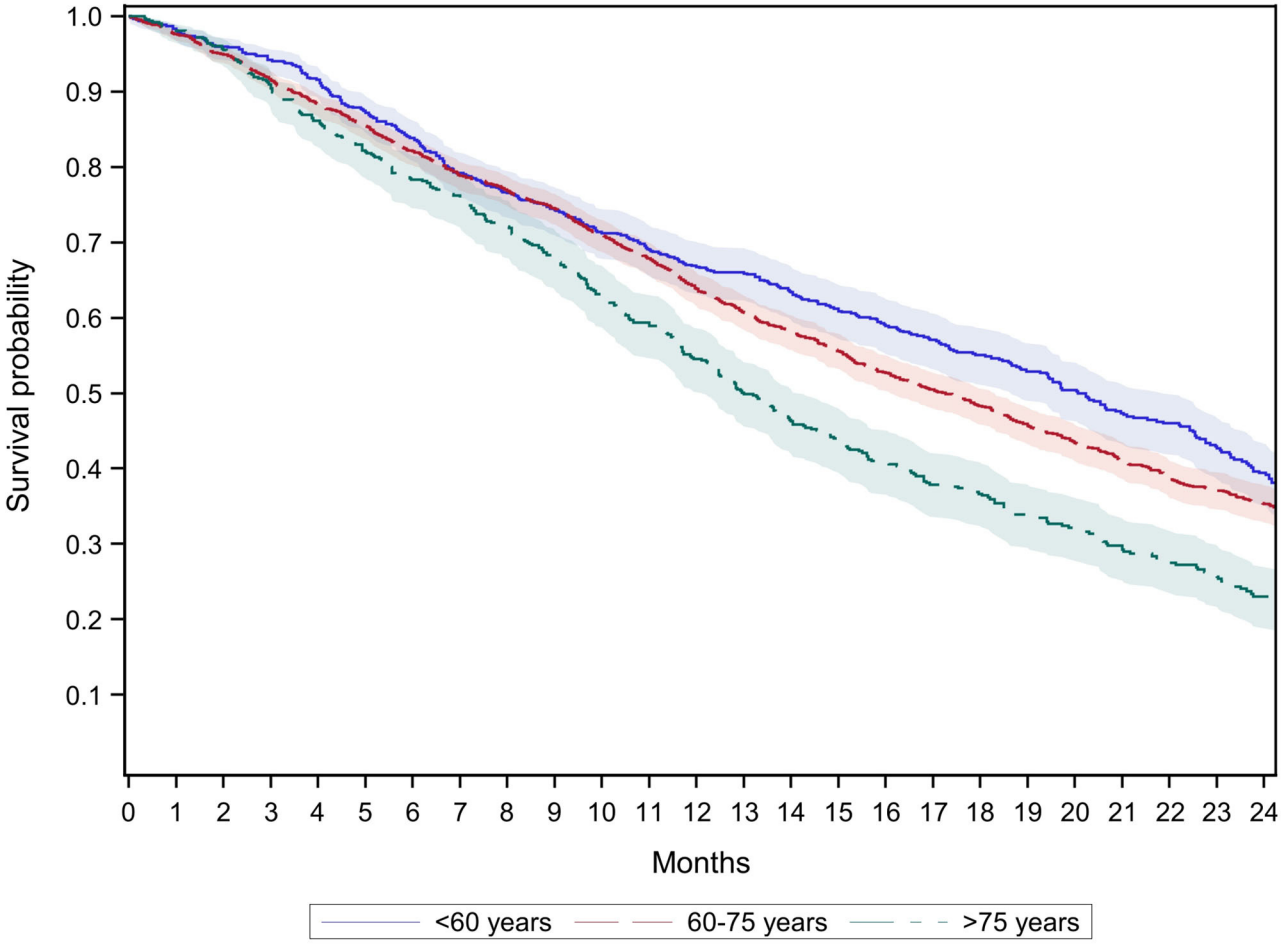
Survival of new users of biologics with colorectal cancer by age groups (GePaRD only).

Stratified by age group (GePaRD only), the 12-month survival among new users of biologics was 7–18% lower in CRC patients of the oldest age group (>75 years) compared to the two younger age groups (<60 and 60–75 years) (Table 3). As shown in Figure 3, the survival curves of the oldest age group started to diverge from the younger age groups after 3 months. The respective confidence intervals were non-overlapping (which corresponds to statistically significant differences) from month 10 onwards. After 24 months, the probability of dying was 61% in the youngest age group (<60 years), 65% in the age group 60–75 years, and 77% in the oldest age group (>75 years).

**Figure 4:**
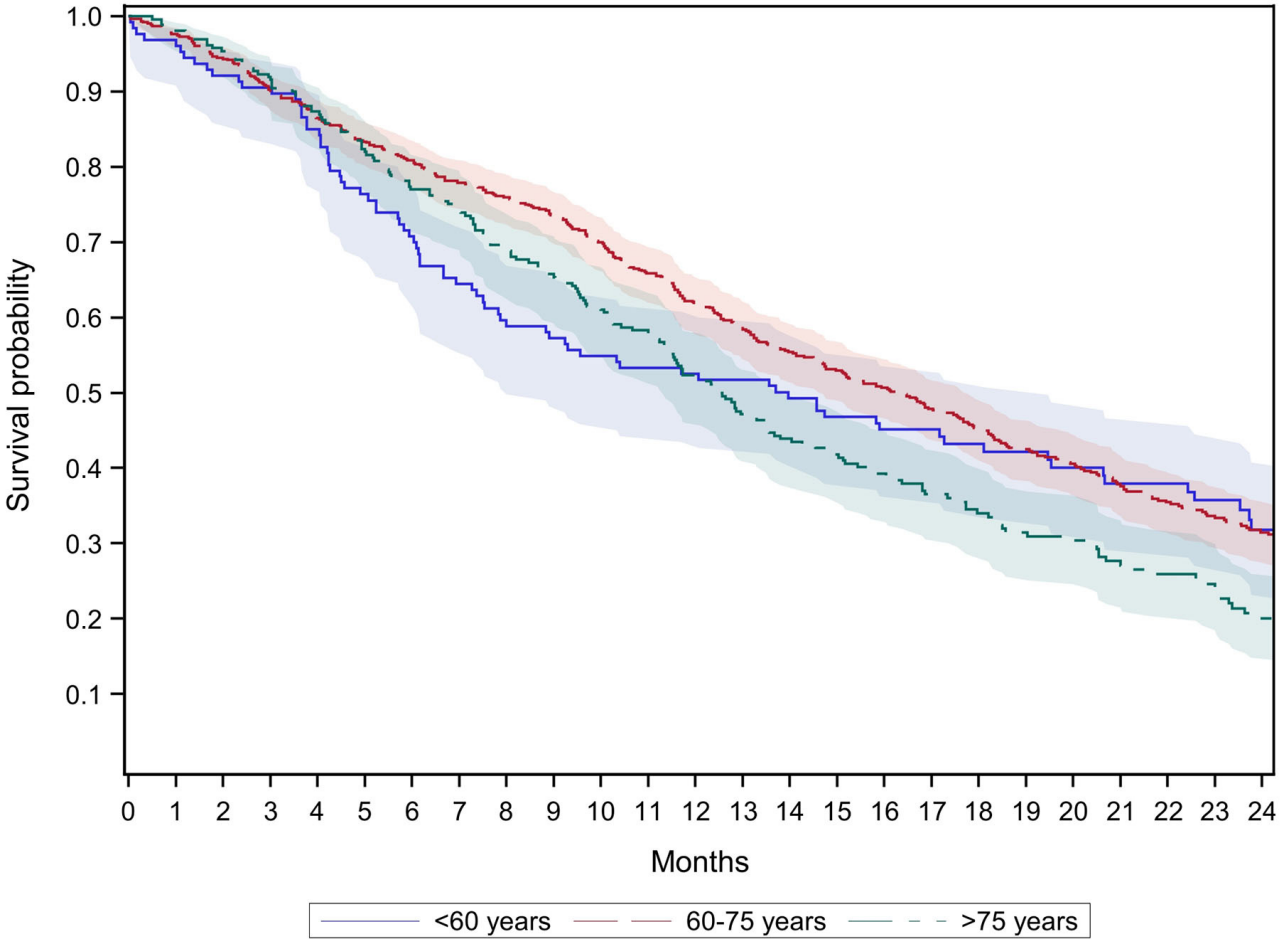
Survival of new users of biologics with colorectal cancer and selected comorbidities^†^ by age groups (GePaRD only).^†^We considered comorbidities that often led to exclusion of patients from randomized controlled trials investigating biologics in colorectal cancer patients: cardio-vascular diseases, ascites, CNS metastases, bleeding diatheses, and coagulopathy (for a list of the respective ICD-9 and ICD-10 codes see Supplementary Material 3).

Figure 4 shows the results of age-stratified survival analyses restricted to new users of biologics with selected comorbidities (see methods section). In the youngest age group (<60 years), the 12-month survival was 53% and thus 14% lower compared to the survival observed in the unrestricted group of patients aged <60 years (Table 3). The 12-month survival in the youngest age group (<60 years) was similar to the oldest age group (>75 years) and lower compared to age group 60–75 years. Between months 6 and 12, the differences in the survival curves between the youngest age group and the age group 60–75 years were statistically significant (non-overlapping 95% confidence intervals). After 12 months, the survival probability in the youngest age group approached the survival probability in the age group 60–75 years. After 24 months, the survival probabilities were 32% in the two younger age groups (<60 years and 60–75 years) and 20% in the oldest age group (>75 years).

## Discussion

This observational population-based study of more than 4,500 CRC patients from three European countries showed that CRC patients treated with biologics in the real-world setting differ substantially from those included in pivotal RCTs of those drugs. In particular, the CRC patients in the real-world setting were older and had comorbidities used as exclusion criteria in the RCTs. This might explain the relatively poor absolute survival observed in our study. Furthermore, we observed different patterns regarding utilization of the EGFR-inhibitors cetuximab vs. panitumumab between countries.

**Figure 5:**
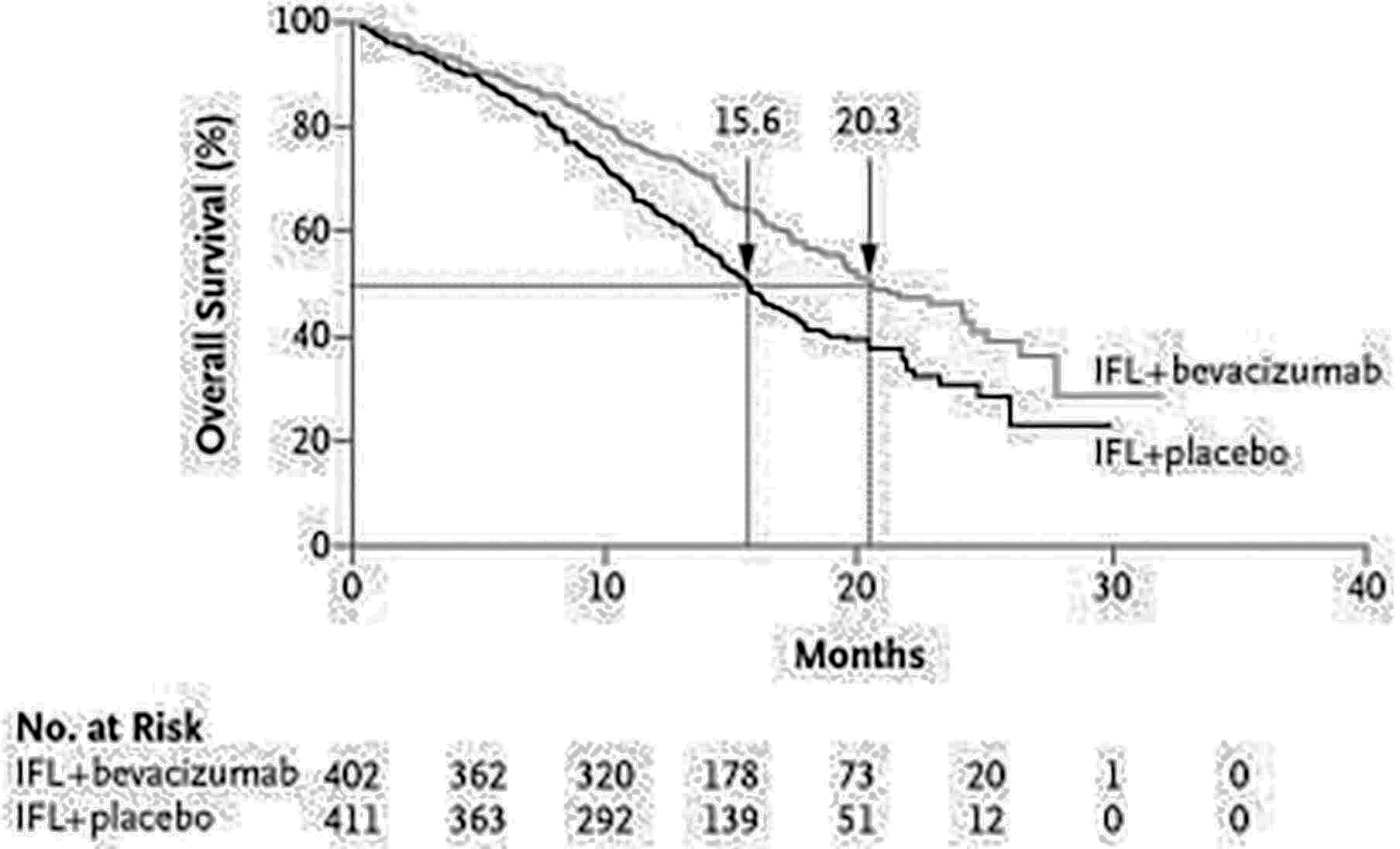
For comparison: Survival of users of standard chemotherapy and bevacizumab and survival of users of standard chemotherapy alone as found in the RCT by Hurwitz et al. (5).

In GePaRD, where survival probability could be estimated more precisely than in the other databases, a 12-month absolute survival of 60–62% was observed among new users of biologics, also in analyses restricted to new users of bevacizumab (Table 3). By contrast, the 12-month survival in the RCT by Hurwitz et al. was 74% for bevacizumab users (Figure 5) and thus considerably higher than in GePaRD (5). Also, a review of 17 RCTs investigating the role of biologics combined with standard chemotherapy as first-line treatment of CRC reported 12-month survival rates higher to our findings for the vast majority of studies: Survival was higher in 16 RCTs, and in one RCT it was either higher or similar, depending on the respective chemotherapy (3). The difference in survival—as assessed by indirect comparison—would have even been larger if the sex distribution in GePaRD (unusually high proportion of female CRC patients) had been similar to the RCTs, keeping in mind that partly higher relative survival rates have been reported for female CRC patients compared to male patients (23).

To explore potential reasons for the observed survival rates, further factors need to be taken into account. The patients in our study were about 6 years older as compared to the patients of the pivotal RCT on bevacizumab (5). Also, most of the other RCTs reviewed by Mahipal and Grothey reported a lower median age compared to our study population. Not surprisingly, our analyses stratified by age showed the lowest absolute survival for the oldest age group (>75 years). Similarly, a study analyzing data from four RCTs found a lower 12-month survival for patients >70 years compared to younger patients (24). Thus, the age structure is likely an important factor explaining the relatively poor 12-month survival observed in our study. Interestingly, our subgroup analysis focusing on CRC patients <60 years with comorbidities showed a 12-month survival of only 50%, suggesting that presence of comorbidities is another important predictor of 1-year mortality among CRC patients treated with biologics. Half of the 17 RCTs reviewed by Mahipal and Grothey, including Hurwitz et al., excluded a priori patients with an Eastern Cooperative Oncology Group (ECOG) performance status of ≥2. We could not assess the ECOG performance status in our study but there were patients in our cohort with comorbidities often used as exclusion criteria in the RCTs. Given these comorbidities and the older age in our cohort, we assume that the ECOG status in the real-world setting is worse than in RCTs. Furthermore, the use of biologics as first vs. second line therapy may be considered in the interpretation of our findings. While all RCTs reviewed by Mahipal and Grothey investigated the effectiveness of biologics as first line therapy, studies published in 2013 also suggested a benefit as second line therapy (i.e., continuation beyond first tumor progression) (25). This may have influenced clinical practice, but we did not find differences in survival between the cohorts 2010 vs. 2014, nor did we observe a relevant difference between new users of biologics vs. prevalent users. Overall, it seems that CRC patients receiving biologics in the real-world setting mainly differ from those enrolled in RCTs of biologics with respect to the presence of comorbidities and age distribution.

The comparison of our findings to other studies using real-world data from Europe is hampered regarding studies using certain criteria to select patients, e.g., if they excluded patients with early disease progression (11, 12) or focused on patients with metachronous metastases (13). A study from the UK based on medical records, which included unselected patients with advanced CRC (*N* = 714) similar to our approach, confirmed our findings. They found a 12-month survival of ~66% in patients who received bevacizumab with the first-line chemotherapy (26). The median age of the study population was similar to our study population and the authors reported comorbidities such as hypertension in 21–34% of the patients and cardiac disorders in 3–7%. Future research focusing on the comparison of patients' characteristics in different countries would be of great interest.

Interestingly, we observed some differences between countries in our analyses. Survival of CRC patients in the German database (GePaRD) was partly statistically significantly lower as compared to the Dutch database (PHARMO), which could not be explained by differences in the age and sex distribution. The prevalence of comorbidities was lower in Dutch patients than in German patients. In part, we assume this resulted from differences in the coding practice but it might also indicate a less selective use of biologics in Germany than in the Netherlands. In other words, as compared to Dutch CRC patients, German patients might be more likely to receive biologics despite an already very poor prognosis or an increased risk of biologic-related adverse reactions due to comorbidities. There were also differences in utilization patterns between countries. Unlike in the German or Italian database, the EGFR-inhibitor cetuximab hardly played a role in the Netherlands, while panitumumab, another EGFR-inhibitor, was used in about one quarter of patients in the Netherlands and in Germany in 2010 but hardly played a role in Italy. Given that all drugs are authorized by the European Medicines Agency, these differences cannot be explained by marketing authorizations. Instead, country-specific reimbursement practices, costs, marketing strategies, or different clinical practices might explain the observed patterns.

It is beyond the scope or the possibilities of our study to judge whether the current use of biologics in the real-world setting is appropriate and clinically justified in all patients receiving biologics. It should still be noted that the risk-benefit ratio of these drugs, as investigated in RCTs, could easily get out of balance if comorbidities increased the risk of severe adverse events or if advanced age or poor prognosis (i.e., terminal illness) lowered the potential benefit on survival. Indeed, our study's findings regarding age, comorbidity, and survival among users of biologics in the real-world setting support concerns that the risk-benefit ratio might be less favorable than in RCTs. Of note, this does not question the efficacy of the drugs regarding tumor progression but solely refers to the selection of patients receiving these drugs. Critical evaluation of treatment decisions regarding biologic use in CRC patients is therefore required, also taking into account ethical issues, e.g., prescribing drugs to patients with poor prognosis where risks may outweigh benefits.

To the best of our knowledge, this is the largest study providing real-world evidence on CRC patients using biologics in Europe and the only study conducting parallel analyses based on databases from different European countries. The study illustrates that large source populations are indeed needed to address research questions on this rare exposure. The sample size in the Dutch and the Italian database was still relatively small but patterns in utilization could be assessed anyhow. The confidence intervals of survival estimates were rather large in these two databases but still partly non-overlapping (e.g., survival curves in PHARMO vs. GePaRD). The databases used for our study also have limitations. The coding of diagnoses is often suboptimal in such databases and coding practices could differ between countries. We assume that this explains the heterogeneous and partly low proportion of CRC patients with codes for metastases as it is very unlikely that these drugs are used “off-label” in non-metastatic CRC patients. Also with respect to comorbidities there was variation in the prevalence between databases, which may in part be explained by these coding issues. Information on molecular subtypes, especially regarding the KRAS status, would have been interesting for additional analyses but was not available in the data used for this study. The same applies to information on the concomitant treatment with chemotherapy. The role of specific cytostatic agents investigated in trials, e.g., the use of capecitabin and bevacizumab in elderly patients in the real-world setting could thus not be assessed in our study (27). We could only do some analysis on concomitant chemotherapy. For example, an analysis in a subsample of new users of GePaRD for whom information on the number of cytostatic agents was available suggested a very high proportion of patients receiving concomitant chemotherapy. In addition, we focused on patterns of use and absolute survival in our study while studies based on primary data often additionally assessed progression-free survival. Although progression-free survival might be assessable with secondary data as well, there is more uncertainty as compared to absolute survival. Also, the follow-up in our data was limited in the 2014 cohorts due to the lag in data availability. Finally, it was beyond our scope to assess treatment regimens (duration, dose, treatment lines) of biologics, which would have required further assumptions and would have been difficult to harmonize across databases. Our study was merely descriptive and focused on patients receiving biologics. Comparison of survival to a control group not receiving biologics would be highly problematic due to confounding by indication and unmeasured confounders.

In conclusion, our study illustrated the potential of European healthcare databases for the real-world monitoring of biologics in the treatment of CRC. These databases do not represent the ideal of a homogeneous and complete European cancer registry with detailed, high-quality data on patient- and tumor-related factors and treatment. As long as such a registry does not exist, we feel it is important to use the specific potential of existing databases in order to allow the various pieces of evidence to complement each other. Consistently across databases, our findings suggest that in the real-world setting, CRC patients treated with biologics are older and have a higher burden of comorbidities as compared to CRC patients enrolled in RCTs of biologics. This may explain the relatively poor 12-month survival rate observed in our study. Our findings highlight the importance of carefully evaluating and reflecting clinical decision making when treating CRC patients with biologics in the real-world setting given that the risk-benefit ratio may vary depending on age and co-existing conditions.

## Data Availability Statement

The datasets presented in this article are not readily available because PHARMO: The datasets generated and/or analyzed during the current study are not publicly available due to privacy reasons. University of Messina/Caserta LHU: The datasets generated and/or analyzed during the current study are not publicly available due to privacy reasons. GePaRD: In accordance with German data protection regulations, access to the data of this study may only be given to third parties within the realm of collaborations with BIPS and after signing an agreement for guest researchers. As we are not the owners of the data, we are not legally entitled to grant access to GePaRD. Requests to access the datasets should be directed to Katja A. Oppelt, oppelt@leibniz-bips.de.

## Ethics Statement

Ethical review and approval was not required for the study on human participants in accordance with the local legislation and institutional requirements. Written informed consent for participation was not required for this study in accordance with the national legislation and the institutional requirements.

## Author Contributions

KO, JK, YI, VI, RH, GT, and UH have made substantial contributions to conception and design of the study and interpretation of data. MT (Caserta LHU), UH (GePaRD), and RH (PHARMO) have made substantial contributions to the acquisition of data. KO, JK, YI, and VI have made substantial contributions to the analysis of data. KO and UH have been involved in drafting the manuscript. JK, YI, VI, RH, MT, GT, and UH have critically revised the manuscript for important intellectual content. All authors have given final approval of the version to be published and agreed to be accountable for all aspects of the work.

## Conflict of Interest

GT has served as a speaker, a consultant, and an advisory board member for Mylan, Sanofi, GSK, Biogen, Ipsen, Shire, AstraZeneca, Eli Lilly, and has received research funding from Amgen, AstraZeneca, Daiichi Sankyo, Boehringer. The remaining authors declare that the research was conducted in the absence of any commercial or financial relationships that could be construed as a potential conflict of interest.
