# Supplementary material for: Characteristics and Absolute Survival of Metastatic Colorectal Cancer Patients Treated With Biologics: A Real-World Data Analysis From Three European Countries
Source: Front Oncol. 2021 Mar 5;11:630456. doi: 10.3389/fonc.2021.630456 (PMC7973261; doi:10.3389/fonc.2021.630456)
Supplement: Supplementary file 1 [file Data_Sheet_1.docx]

Supplementary Material 1

# Database descriptions

## Caserta LHU

Caserta LHU is a record linkage database containing claims data from several databases. Started in 2009, it covers around 1.2 million residents in Caserta, Italy. Collected data include: 1) demographic registry that contains information about the date of birth, gender, date of registration in the regional healthcare system, and where applicable, date of death or deregistration; 2) drug dispensing database, that contains data on the date of dispensing, number and cost of dispensed packages, active substance and brand name, coded with ATC code and AIC (i.e. Italian market authorization code) code; 3) hospital discharge database, containing information on date of hospital admission and discharge, diagnosis-related group (DRG), primary diagnosis and up to five secondary diagnoses and performed hospital procedures coded with ICD9-CM codes; 4) referrals for outpatient diagnostic tests and specialist’s visits database, including test or visit specific code, date of test and name of laboratory where the test is carried out; 5) registry for exemption code-granted patients, that contains coded information about chronic diseases or socioeconomic factors; 6) emergency department admissions database, including reasons for admission to emergency departments, as well as date of admission and discharge. Drug dispensing in the hospital or drugs dispensed over the counter (OTC) are not traced.

All databases can be linked through an anonymous subject identifier. For a subsample of 60% linked data to prescriptions of general practitioners is available. Further included information are electronic therapeutic plans for specific drugs, results of performed diagnostic tests, geriatric evaluation forms starting in 2013, and data from a diabetes and a hypertension registry starting in 2015.

## GePaRD

GePaRD is based on claims data from four statutory health insurance providers in Germany and currently includes information on approximately 25 million persons who have been insured with one of the participating providers since 2004 or later. In addition to demographic data, GePaRD contains information on drug dispensations, outpatient and inpatient services and diagnoses. Per data year, there is information on approximately 17% of the general population and all geographical regions of Germany are represented.

For this study, we used diagnosis codes that are registered in GePaRD according to the International Statistical Classification of Diseases and Related Health Problems, 10th revision, German Modification (ICD-10 GM). With respect to the inpatient setting, we considered main and secondary hospital discharge diagnoses, but not admission diagnoses. We only considered diagnoses from the outpatient setting coded as “confirmed”. We further used dispensations codes from the out- and inpatient sector. In the outpatient sector, dispensation information is registered in a specific code (Pharmazentralnummer, PZN) identifying the active agent, the compounding, the producing company, and the packaging size and is retrieved via ATC codes. Information on dispensations in the inpatient sector is based on the German modification of the International Classification of Procedures in Medicine (ICPM), the Operationen- und Prozedurenschlüssel (OPS).

## PHARMO Database Network

The PHARMO Database Network is a population-based network of electronic healthcare databases currently covering over 6 million active persons out of 17 million inhabitants of the Netherlands. It enables to follow-up more than 9 million persons of a well-defined population in the Netherlands for an average of twelve years. PHARMO combines anonymous data from different primary and secondary healthcare settings in the Netherlands. For this study, we used data from the Hospital Database, In-patient Pharmacy Database and Out-patient Pharmacy Database. All three database are linked on a patient-level. The Hospital Database comprises hospital admissions for more than 24 hours and admissions for less than 24 hours for which a bed is required (i.e. in-patient records). The records include information on hospital admission and discharge dates, discharge diagnoses and procedures. Diagnoses are coded according to the WHO International Classification of Diseases (ICD). The In-patient Pharmacy Database comprises drug dispensings from the hospital pharmacy, given during a hospitalization or for which a bed is required (i.e. biologics given intravenous). The Out-patient Pharmacy Database comprises GP or specialist prescribed healthcare products dispensed by the (hospital-based) out-patient pharmacy. Since 2012, high budget impact medication including biologics cannot be dispensed by community pharmacies and are dispensed by hospital-based out-patient pharmacies. The dispensing records include information on type of product, date, strength, dosage regimen, quantity, route of administration, prescriber specialty and costs. Drug dispensings are coded according to the WHO Anatomical Therapeutic Chemical (ATC) Classification System.
