# Supplementary material for: Characteristics and Absolute Survival of Metastatic Colorectal Cancer Patients Treated With Biologics: A Real-World Data Analysis From Three European Countries
Source: Front Oncol. 2021 Mar 5;11:630456. doi: 10.3389/fonc.2021.630456 (PMC7973261; doi:10.3389/fonc.2021.630456)
Supplement: Supplementary file 2 [file Data_Sheet_2.docx]

Supplementary Material 2

# ATC codes for biologics considered

| **Substance name** | **ATC code** |
| --- | --- |
| Bevacizumab | L01XC07 |
| Cetuximab | L01XC06 |
| Panitumumab | L01XC08 |
