# Supplementary material for: Characteristics and Absolute Survival of Metastatic Colorectal Cancer Patients Treated With Biologics: A Real-World Data Analysis From Three European Countries
Source: Front Oncol. 2021 Mar 5;11:630456. doi: 10.3389/fonc.2021.630456 (PMC7973261; doi:10.3389/fonc.2021.630456)
Supplement: Supplementary file 3 [file Data_Sheet_3.docx]

Supplementary Material 3

# ICD-9 and ICD-10 codes for defining comorbidity

| **Comorbidity** | **ICD-9** | **ICD-10** | **Description of the code** |
| --- | --- | --- | --- |
| Cardio-vascular diseases | | | |
|  | 40201, 40211, 40291 | I110 | Hypertensive heart disease with (congestive) heart failure |
|  | 40301, 40311, 40391 | I120 | Hypertensive renal disease with renal failure |
|  | 40401, 40411, 40491 | I130 | Hypertensive heart and renal disease with (congestive) heart failure |
|  | 40400, 40410, 40490, 40402, 40412, 40492 | I131 | Hypertensive heart and renal disease with renal failure |
|  | 40403, 40413, 40493 | I132 | Hypertensive heart and renal disease with both (congestive) heart failure and renal failure |
|  | 4111, 4131, 4139 | I20 | Angina pectoris |
|  | 4106%, 41091, 41071, 4109% | I21 | Acute myocaridal infarction |
|  | 41001, 41011, 41021, 41031, 41041, 41051, 41061, 41081, 41091 | I22 | Subsequent myocardial infarction |
|  | 42979, 42971, 42971, 42979, 4295, 4296, 42979, 42979 | I23 | Certain current complications following acute myocardial infarction |
|  | 41181, 4110, 41189, 41189 | I24 | Other acute ischaemic heart diseases |
|  | 4140, 41401, 4292, 412, 41410, 41419, 41411, 4148, 4149 | I25 | Chronic ischaemic heart disease |
|  | 4231, 4232, 4230, 4239, 4238, 4239 | I31 | Other diseases of pericardium |
|  | 42099, 4200 | I32 | Pericarditis in diseases classified elsewhere |
|  | 4240 | I34 | Nonrheumatic mitral valve disorders |
|  | 4241 | I35 | Nonrheumatic aortic valve disorders |
|  | 4243 | I37 | Pulmonary valve disorders |
|  | 42490, 42499 | I38 | Endocarditis, valve unspecified |
|  |  | I390 | Mitral valve disorders in diseases classified elsewhere |
|  |  | I391 | Aortic valve disorders in diseases classified elsewhere |
|  |  | I393 | Pulmonary valve disorders in diseases classified elsewhere |
|  |  | I394 | Multiple valve disorders in diseases classified elsewhere |
|  | 42491 | I398 | Endocarditis, valve unspecified, in diseases classified elsewhere |
|  | 4254, 42511, 42518, 4250, 4253, 4255, 4259, 4252 | I42 | Cardiomyopathy |
|  | 4257, 4258 | I43 | Cardiomyopathy in diseases classified elsewhere |
|  | V1253, 4275 | I46 | Cardiac arrest |
|  | 4271 | I470 | Re-entry ventricular arrhythmia |
|  | 4270 | I471 | Supraventricular tachycardia |
|  | 4271 | I472 | Ventricular tachycardia |
|  | 42731 | I48 | Atrial fibrillation and flutter |
|  |  | I490 | Ventricular fibrillation and flutter |
|  | 42781 | I495 | Sick sinus syndrome |
|  | 42789 | I498 | Other specified cardiac arrhythmias |
|  | 4279 | I499 | Cardiac arrhythmia, unspecified |
|  | 4280, 4281, 4289 | I50 | Heart failure |
|  | 42971, 4295, 4296, 42989, 4290, 4291, 4292, 4293, 4299 | I51 | Complications and ill-defined descriptions of heart disease |
|  | 4321, 4320, 4329 | I62 | Other nontraumatic intracranial haemorrhage |
|  | 433%, 43401, 43491 | I63 | Cerebral infarction |
|  |  | I64 | Stroke, not specified as haemorrhage or infarction |
|  | 4332, 43300, 4331, 4333, 43380, 43390 | I65 | Occlusion and stenosis of precerebral arteries, not resulting in cerebral infarction |
|  | 43400, 43410, 43490 | I66 | Occlusion and stenosis of cerebral arteries, not resulting in cerebral infarction |
|  | 44329, 4373, 4370, 0463, 4372, 4375, 4376, 4374, 4379 | I67 | Other cerebrovascular diseases |
|  | 4378, 4374 | I68 | Cerebrovascular disorders in diseases classified elsewhere |
|  |  | I69 | Sequelae of cerebrovascular disease |
|  | 5723 | K766 | Portal hypertension |
| Ascites | | | |
|  | 7895% | R18 | Ascites |
| Bleeding diatheses | | | |
|  | 2870 | D690 | Allergic purpura |
|  | 2871 | D691 | Qualitative platelet defects |
|  | 2878 | D698 | Other specified haemorrhagic conditions |
|  | 2879 | D699 | Haemorrhagic condition, unspecified |
|  | 2867 | D683 | Haemorrhagic disorder due to circulating anticoagulants |
| Coagulopathy | | | |
|  | 2866 | D65 | Disseminated intravascular coagulation [defibrination syndrome] |
|  | 2860 | D66 | Hereditary factor VIII deficiency |
|  | 2861 | D67 | Hereditary factor IX deficiency |
|  | 2864, 2862, 2863, 2865, 2867, 2869 | D68 | Other coagulation defects |
| CNS metastases | | | |
|  | 1983 | C793 | Secondary malignant neoplasm of brain and cerebral meninges |
|  | 1984 | C794 | Secondary malignant neoplasm of other and unspecified parts of nervous system |
